# Archaea Dominate the Ammonia-Oxidizing Community in Deep-Sea Sediments of the Eastern Indian Ocean—from the Equator to the Bay of Bengal

**DOI:** 10.3389/fmicb.2017.00415

**Published:** 2017-03-16

**Authors:** Jing Wang, Jinjun Kan, Xiaodong Zhang, Zhiqiang Xia, Xuecheng Zhang, Gang Qian, Yanyi Miao, Xiaoyun Leng, Jun Sun

**Affiliations:** ^1^College of Marine and Environmental Sciences, Tianjin University of Science and TechnologyTianjin, China; ^2^Tianjin Key Laboratory of Marine Resources and Chemistry, Tianjin University of Science and TechnologyTianjin, China; ^3^Stroud Water Research CenterAvondale, PA, USA

**Keywords:** Eastern Indian Ocean, deep-sea sediment, ammonia-oxidizing archaea and bacteria (AOA and AOB), nitrogen cycle, clone library, qPCR

## Abstract

Ammonia-oxidizing Archaea (AOA) and ammonia-oxidizing Bacteria (AOB) oxidize ammonia to nitrite, and therefore play essential roles in nitrification and global nitrogen cycling. To better understand the population structure and the distribution of AOA and AOB in the deep Eastern Indian Ocean (EIO), nine surface sediment samples (>3,300 m depth) were collected during the inter-monsoon Spring 2013. One sediment sample from the South China Sea (SCS; 2,510 m) was also included for comparison. The community composition, species richness, and diversity were characterized by clone libraries (total 1,238 clones), and higher diversity of archaeal *amo*A genes than bacterial *amo*A genes was observed in all analyzed samples. Real time qPCR analysis also demonstrated higher abundances (gene copy numbers) of archaeal *amo*A genes than bacterial *amo*A genes, and the ratios of AOA/AOB ranged from 1.42 to 8.49 among sites. In addition, unique and distinct clades were found in both reconstructed AOA and AOB phylogeny, suggesting the presence of niche-specific ammonia-oxidizing microorganisms in the EIO. The distribution pattern of both archaeal and bacterial *amo*A genes revealed by NMDS (non-metric multidimensional scaling) showed a distinct geographic separation of the sample from the SCS and most of the samples from the EIO following nitrogen gradients. Higher abundance and diversity of archaeal *amo*A genes indicated that AOA may play a more important role than AOB in the deep Indian Ocean. Environmental parameters shaping the distribution pattern of AOA were different from that of AOB, indicating distinct metabolic characteristics and/or adaptation mechanisms between AOA and AOB in the EIO, especially in deep-sea environments.

## Introduction

Nitrification is an integrative component in the nitrogen cycle, which includes oxidization of ammonia to nitrite by ammonia-oxidizing Bacteria (AOB) and ammonia-oxidizing Archaea (AOA), and then nitrite is further oxidized to nitrate by nitrite-oxidizing Bacteria (NOB). As the first and also the rate-limiting step, ammonia oxidation to nitrite is mediated by both AOB and AOA (Norton et al., 2002; Schleper and Nicol, 2010). The chemolithotrophic AOB are placed taxonomically in the *beta*- and the *gamma*-subdivision of Proteobacteria, and their natural population/distribution has been widely investigated by molecular tools, such as using the 16S rRNA gene sequences (Stephen et al., 1996, 1998; Kowalchuk et al., 2000) and the genes encoding ammonia monooxygenase (*amo*A; Bothe et al., 2000; Purkhold et al., 2000). In recent years, novel archaeal groups that function as ammonia oxidizers containing *amo*A genes have also been observed and they may contribute greatly to nitrification processes as well (Venter et al., 2004; Schleper et al., 2005). Environmental AOA sequences are mainly affiliated with several groups, such as marine group I (MGI), putatively marine group pSL12, thermophilic AOA (ThAOA) and soil group I.1b (Durbin and Teske, 2011; Pester et al., 2011; Hatzenpichler, 2012). In order to better understand their roles in global nitrogen cycling, it is critical to characterize the abundance, diversity, and distribution of AOA and AOB in natural environments.

Nitrogen is likely the limiting nutrient in marine ecosystems (Gruber and Sarmiento, 1997), and therefore the nitrogen cycling in the ocean is of particular interest (Deutsch et al., 2007). AOA and AOB have been commonly found in marine sediments all over the world, including the East China Sea (Dang et al., 2008), the South China Sea (Jin et al., 2011), hydrothermal vents of the Pacific Ocean (Wang et al., 2009; Nunoura et al., 2010), the western Pacific Ocean (Cao et al., 2011a), the tropical West Pacific Continental Margin (Dang et al., 2009), the Northeastern Japan Sea (Nakagawa et al., 2007), the Southern North Sea (Lipsewers et al., 2014), and also the deep-sea environments (Nunoura et al., 2013; Xu et al., 2014; Lagostina et al., 2015; Luo et al., 2015). Among these studies, ratios of AOA/AOB gene copy numbers varied from 0.0003 to 232.3, and no consistent patterns have been observed. Obviously the distribution and variations of AOA and AOB are relevant to their metabolic characteristics and their responses to environmental gradients as well. For instance, AOA has been reported to have a higher affinity to ammonium than AOB in oligotrophic environment (Martens-Habbena et al., 2015). The dominant group and distribution of AOB could be driven by salinity, pH, and nutrient availability (Bernhard et al., 2007; Cao et al., 2012; Li et al., 2015), while the distribution of AOA varied along water depth, redox variation, and concentration of ammonium in water column or marine sediments (Wuchter et al., 2006; Dang et al., 2009; Roussel et al., 2009; Flood et al., 2015). Although a global distribution, diverse composition, and high abundance of AOA and AOB have been reported (Rotthauwe et al., 1997; Nicol and Schleper, 2006; Francis et al., 2007; Cavicchioli et al., 2007), compared to other regions, we have very limited knowledge on these microorganisms in the Indian Ocean.

The Indian Ocean is the third largest ocean in the world, which is characterized by two semi-enclosed basins in the north, and greatly influenced by seasonal monsoon (Fine et al., 2008; Rixen et al., 2009). Strong stratification induced by monsoon suppresses up welling and mixing of the deep waters, making the Eastern Equatorial Indian Ocean a typical oligotrophic area (Kumar et al., 2009). The Arabian Sea (AS) and the Bay of Bengal (BOB) are the two basins in the north part of the Indian Ocean and are relatively less oligotrophic compared to the Equatorial region. The frequent dust input to the Arabian Sea, enhanced advection, and vertical eddy mixing bring small detritus and nutrients to the Central/Eastern AS (McCreary et al., 1993). In contrast, lack of detritus transport limits organic matter accumulation in the BOB (Kumar et al., 2004), meanwhile, river runoff, evaporation, seasonal advection, and mixing result in low surface salinity and high surface stratification (George et al., 1994; Kumar et al., 2002). The oceanic circulation and heat storage variation caused by low surface salinity strengthens the vertical stratification (Nyadjro et al., 2013). Finally, the BOB is also characterized by its low productivity (Madhupratap et al., 2003; Kumar et al., 2004) and high nitrification rate (Srinivas et al., 2011). Remineralization that consumes oxygen in the BOB is also weaker, making the BOB less hypoxic than AS, but still stronger than the Equator and other areas in the Eastern Indian Ocean (EIO; Kumar et al., 2002).

The Indian Ocean and sediments are highly oligotrophic, and most of the areas including the BOB are covered by volcanogenic sediments that are characterized by high illite and chlorite in the clay mineral assemblages (Venkatarathnam and Biscaye, 1973; Madhupratap et al., 2003). River borne solids with terrestrial organic carbon (Goldberg and Griffin, 1970; Fontugne and Duplessy, 1986) also contribute to the surface sediments in the BOB, where bacterial oxidation and respiration occurred (Peterson and Prell, 1985; Middelburg, 1989; Walsh, 2014). Oxidation and reduction of organic matter in the deep-sea sediments are active for resource and energy flow, which provide ideal environments for microbial nitrogen transformations. However, no direct measurements were carried out on the nitrification/denitrification processes in sediment of the BOB. The diversity and distribution of bacterial communities in the water column of the BOB and Equatorial area of the EIO has been screened by a high throughput sequencing analysis (Wang et al., 2016), but the details of the composition, diversity and distribution of nitrogen transforming microbes in these areas still remain largely unknown.

In this study, deep-sea sediments (>3,300 m) were sampled to investigate the diversity and distribution of archaeal and bacterial *amo*A genes. Clone library and q-PCR analysis were applied to characterize the community composition and relative abundances of ammonia oxidizing genes (*amo*A). Based on the multivariate statistics and phylogenetic analysis, diversity and distribution patterns of AOA and AOB in the EIO were compared and discussed. Environmental factors driving the distributions were also analyzed by redundancy analysis (RDA).

## Methodology

### Sample collection and environmental parameter measurements

**Figure 1:**
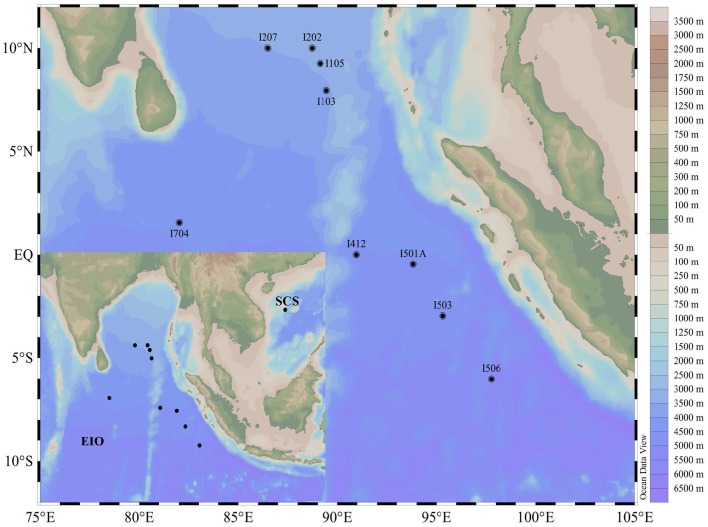
**Map showing the sampling sites in the Eastern Indian Ocean (EIO) and the South China Sea (SCS)**.

Sediment samples were collected during an open cruise supported by the National Natural Science Foundation of China in the EIO aboard the R/V “*Shiyan* 1” from March 22nd to April 18th in 2013. Nine stations were selected and sediment samples were collected along five main transections targeting the Bay of Bengal (I1 and I2), the Equator area (I4 and I7), and the transection (I5) paralle to the coastline of the Sumatra (the Eastern Boundary of the EIO; Figure 1). Sediment samples were collected by a gravity sampler with a temperature probe attached. The surface of the sediment column was sealed with seawater until release from the gravity sampler. After removing the top 5 cm surface sediment that might be disturbed during sampling, the sub-samples were taken from the 2 to 5 cm of the column surface. Sub-samples were collected in sterile plastic bags for microbial study and stored in liquid nitrogen immediately. Sediment samples were transported on dry ice and stored in a −80°C freezer until further analysis. For comparison purpose, one sediment sample from the South China Sea (SCS) was taken on March 20th 2013 following the same protocol.

Sedimental porewater was collected for measurement of chemical parameters. Total carbon (TC) and total nitrogen (TN) contents were measured with a CHNSO elemental analyzer (Costech ECS 4010, Italy). The contents of nitrite, nitrate and ammonium in sediments were measured by an AA3 HR analyzer (SEAL Analytical, USA). Detailed site descriptions including time, water depth, location etc. were listed in Table 1.

**Table 1:** **Site description and chemical measurements**.

**Site and Sample information**	**I506**	**I503**	**I501A**	**I412**	**I704**	**I207**	**I202**	**I105**	**I103**	**SCS**
**Stations**										
Sampling time	2013/3/22	2013/3/24	2013/3/26	2013/3/27	2013/4/16	2013/4/27	2013/4/28	2013/4/29	2013/4/30	2013/3/20
Water depth (m)	5,725	4,810	4,527	4,532	4,426	3,515	3,386	3,437	3,614	2,510
Longtitude	97.76754667	95.317495	93.81465	90.95521667	82.02821667	86.49836667	88.72803333	89.12828333	89.44266667	112.8058167
Latitude	−6.019785	−2.957325	−0.464666667	0.00823333	1.554216667	10.00263333	10.00371667	9.261833333	7.943716667	15.69273333
Water Content (%)	77.30	78.26	60.81	59.36	64.94	60.65	64.68	61.43	62.00	55.28
Total Nitrogen (%)	0.1286^**^	0.0707	0.1568^**^	0.1626^**^	0.1519^**^	0.1574^**^	0.1413^**^	0.135^**^	0.133^**^	0.1901^**^
Total Carbon (%)	0.6658	0.6304	5.4087^*^	8.8089^**^	2.3842	9.9288^**^	10.4103^**^	11.6196^**^	11.8085^**^	8.5872^**^
${\text{NO}}_{2}^{-}$ (mg/L)	0.0001	0.0001	0.0003^**^	0.0003^**^	0.0003^**^	0.0003^**^	0.0002^*^	0.0004^**^	0.0003^**^	0.0003^**^
${\text{NO}}_{3}^{-}$ (mg/L)	0.0024	0.0019	0.0034^**^	0.0037^**^	0.0017	0.0042^**^	0.003^**^	0.0034	0.0035^**^	0.0028^*^
${\text{NH}}_{4}^{+}$ (mg/L)	0.0020	0.0019	0.0029^**^	0.0036^**^	0.0035^**^	0.0045^**^	0.0035^**^	0.0038^**^	0.0039^**^	0.0037^**^

*Detection limits for ${\text{NO}}_{2}^{-}$ and ${\text{NO}}_{3}^{-}$ are 0.0001 mg/L, for ${\text{NH}}_{4}^{+}$ is 0.0004 mg/L*.

Differences between data set of each parameter are determined by t-test, significance is represented by P value.

* P < 0.05;

** *P < 0.01*.

### DNA extraction and clone library construction

#### DNA extraction

Approximately 0.5 g (wet weight) of sediment was transferred into a sterile 1.5 ml centrifuge tube. Total genomic DNA was extracted using the PowerSoil Isolation Kit (MoBio, Carlsbad, CA, USA) according to manufacturer's instructions. Dilution was applied when necessary. All DNA extracts were stored at −20°C for further analyses.

#### PCR amplification of *amo*A genes

Primers Arch-amoAF, Arch-amoAR and amoA-1F, amoA-2R were used to amplify archaeal and bacterial *amo*A genes, respectively (Rotthauwe et al., 1997; Francis et al., 2005). PCR amplification was performed in a 25-μl reaction volume containing 0.25 μM each primer, 0.5 U DNA polymerase (Promega, Madison, WI, USA), 2.5 μl 10 × GoTag® Flexi Buffer, 50 mM MgCl_2_ solution, 500 μM (PCR NucleotideMix, 10 mM each) each deoxynucleoside triphosphate, 2.5 μl 0.1% BSA, and 25 ng of template DNA to a final volume of 25 μl. Amplification was performed with the MJ Research PTC-200 Peltier Thermal Cycler (Waltham, MA, USA). The thermal profile used for amplification of the archaeal *amo*A gene followed Wang et al. (2014), which included 5 min at 94°C, followed by 40 cycles of 45 s at 94°C, 30 s at 57°C, and 1 min at 72°C, and a final extension of 7 min at 72°C. Amplification of the bacterial *amo*A gene was performed at 94°C for 3 min, followed by 40 cycles of 30 s at 94°C, 30 s at 55°C, and 45 s at 72°C, and a final extension of 7 min at 72°C. For quantitative polymerase chain reaction (q-PCR) analysis, the same pairs of primer set were used with 45 cycles.

#### Clone library construction

Clone libraries of archaeal and bacterial *amo*A gene-amplified products from all the samples were constructed following the previously described protocol (Weidner et al., 1996). In brief, after total DNA extraction and PCR amplification of the *amo*A genes, PCR products were verified for the correct amplification by running a 1% agarose gel in 1 × TAE buffer at 90 V for 30 min. Gel slices containing the target PCR products were excised with a sterilized scalpel and then purified using the AxyPrep DNA Gel Extraction Kit (250-prep, Axygen, US). The size of purified PCR products was confirmed again by running on agarose gel before being ligated into pMD18-T vector (D101A, TaKaRa, Dalian, China) and then cloned into *Escherichia coli* DH5-α cells according to the modified transformation method developed by Mandel and Higa (1970). Colonies were randomly picked from each clone library and verified for correct insertion of DNA fragment by PCR amplification with primer set M13F (5′-GTTTCCCAGTCACGAC-3′) and M13R (5′-TCACA CAGGAAACAGCTATGAC-3′). PCR products from the positive clones were sequenced by BGI, Beijing. DNA sequences were examined and edited using BioEdit (Tom Hall, North Carolina State University, Raleigh, NC, USA) and MEGA, version 5.01.

### Quantitative real-time PCR

The abundances of archaeal and bacterial *amo*A genes were determined in triplicate using an ABI Step One Plus Thermocycler. Quantification of each sample was based on the fluorescent dye SYBR Green I, which binded to the double-stranded DNA during PCR amplification. Reactions were performed in a 25 μl volume containing 1 μl of DNA, 0.15 μM of each primer and 12.5 μl of FastStart Universal SYBR Green Mastermix (Roche Diagnostic GmbH, Mannheim, Germany). Specificity of the amplification product was verified by melting curve analysis and visualized in agarose gels. Plasmid DNA was extracted by Plasmid Kit (Promega, Madison, WI, USA) from the clones containing the correct insertion, and the concentration was measured with an Eppendorf BioPhotometer (Eppendorf AG, Hamburg, Germany). The copy numbers of *amo*A genes were then calculated based on the concentration of plasmid DNA and amplicon size (Okano et al., 2004; He et al., 2007). The plasmids of the *amo*A gene clones with known concentrations were diluted to produce the standard curve over seven orders of magnitude (1.21 × 10^8^ to 1.21 × 10^2^ copies of template for archaeal *amo*A and 1.14 × 10^8^ to 1.14 × 10^2^ copies of template for bacterial *amo*A).

### Phylogenetic analysis

The partial archaeal and bacterial *amo*A sequences recovered from clone libraries of the EIO and SCS were blasted in GenBank using the BLAST (http://www.ncbi.nlm.nih.gov). The most closely related sequences and additional reference sequences were retrieved and subsequently aligned with representative clones in CLUSTALX (version 2.0.11). Phylogenetic neighbor-joining tree were constructed by MEGA [version 5.01]. The tree branch distances represented the substitution rate and the scale bar represented the expected number of changes per homologous position. Cluster stability was assessed by bootstrap analyses based on 1,000 replicates, and the bootstrap values >50% were shown near nodes.

### Data analysis

Similarity at 97% was used as cut-off value in defining OTUs (operation taxonomy unit). The coverage of each clone library was calculated as C = [1 − (n1/N)] · 100, where, n1 is the number of unique OTUs and N is the total number of clones in a library (Dang et al., 2009). Diversity indices of *amo*A gene (Shannon–Wiener H and Simpson D and Chao1) were calculated based on the OTU data. Rarefaction analysis and two non-parametric richness estimators, and the bias-corrected Chao1 (S_Chao1_) were calculated using DOTUR (Schloss and Handelsman, 2005). These diversity indices and richness estimators are useful for comparing the relative complexity of communities and estimating the completeness of sampling (Lozupone et al., 2007). Non-metric multidimensional scaling (NMDS) analysis was applied to characterize the distribution patterns of AOA and AOB. Pairwise similarities/distances were calculated based on relative abundances of each OTU across all the samples analyzed, and NMDS was performed with the multidimensional scaling (MDS) procedure of the SAS System (SAS Institute Inc., 2008). Stress value <0.1 indicated a good ordination with little risk of misinterpretation of the results (Clarke and Ainsworth, 1993). Correlations between *amo*A gene OTU distribution and environmental factors were analyzed with a redundancy analysis (RDA) using CANOCO 4.5 software (ver. 4.5, Microcomputer Power, Ithaca, NY, USA) (Lepš and Šmilauer, 2003). The raw data were processed by a log transformation before running RDA (Lepš and Šmilauer, 2003). RDA was chosen to determine the relationships between AOA and AOB community structures and the environmental factors because the longest gradient in a detrended correspondence analysis was between 3.0 and 4.0 (Lepš and Šmilauer, 2003).

### Nucleotide sequence accession numbers

The archaeal and bacterial *amo*A gene sequences retrieved in this study have been deposited in GenBank under accession numbers KU595584 to KU595656, and KU595657 to KU595711, respectively.

## Results

### Environmental parameters

The Environmental parameters measured in this study were summarized in Table 1. All the sediment samples from the EIO were taken from 3,386 up to 5,725 m depth, and the sample from the SCS was at a depth of 2,510 m. The sample from the SCS exhibited highest value in total nitrogen content, while four samples from the BOB (I103, I105, I202, and I207) showed higher content of total carbon than other samples. Samples from the BOB also contained higher nitrate, nitrite and ammonium in general. The highest concentration of [${\text{NO}}_{2}^{-}$ + ${\text{NO}}_{3}^{-}$] reached 0.0045 mg/L in sample I207, which was located at the BOB, while the lowest concentration of [${\text{NO}}_{2}^{-}$ + ${\text{NO}}_{3}^{-}$] was 0.002 mg/L in samples I704 and I503, which was close to the equator and the Eastern Boundary of the EIO. Not surprisingly, the highest concentration of ${\text{NH}}_{4}^{+}$ was also detected at station I207 (0.0045 mg/L), while the lowest one was at station I503 (0.0019 mg/L).

### Diversity of archaeal and bacterial *amo*A genes in the eastern indian ocean

**Figure 2:**
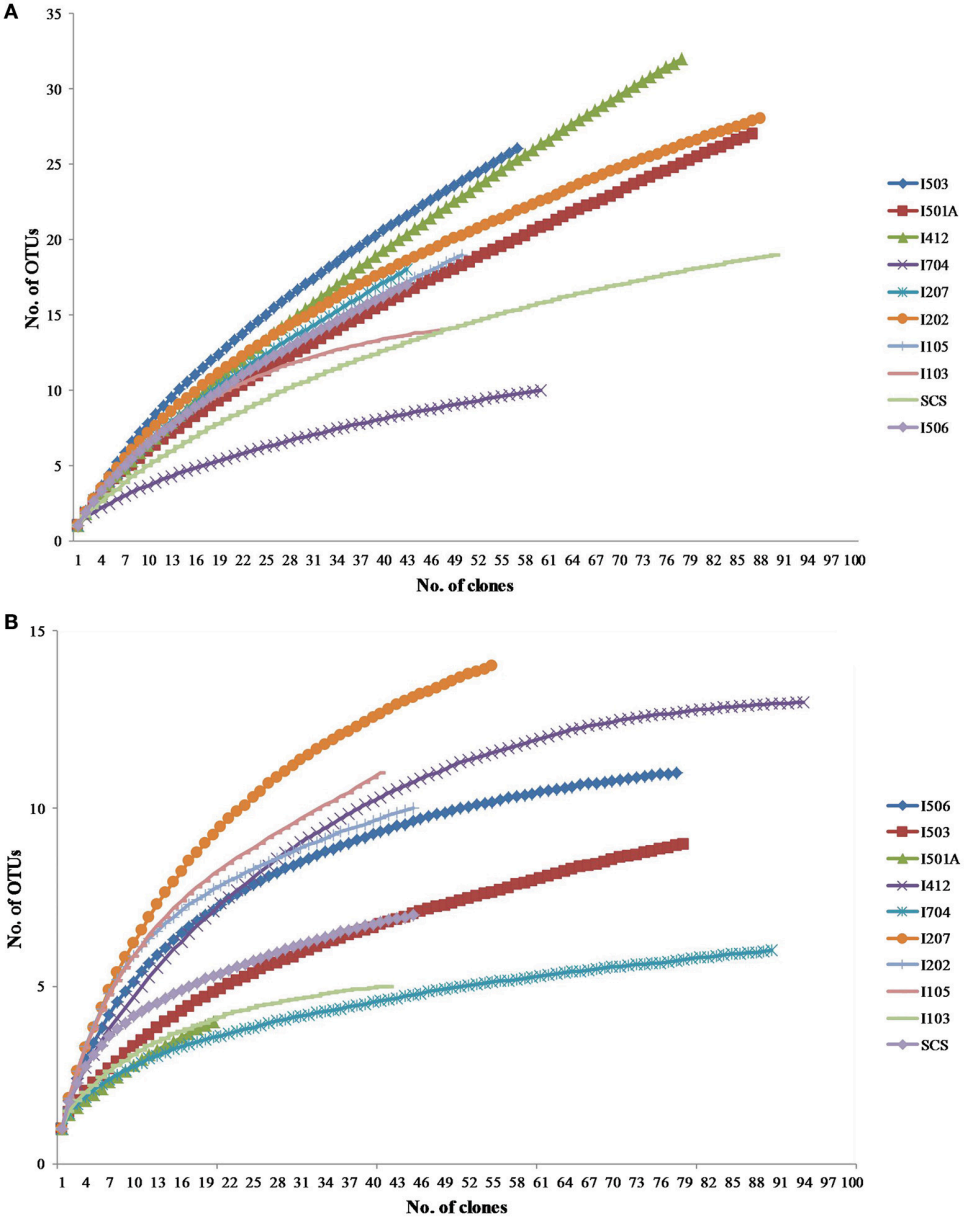
**Rarefaction curves for archaeal (A)** and bacterial **(B)** *amo*A gene clone libraries generated from DOTUR analysis.

Total 20 clone libraries were constructed for archaeal and bacterial *amo*A genes. In 10 libraries of AOA, total 650 clones were sequenced and 53 OTUs were identified at a cut-off value of 97% nucleotide similarity; while for AOB, 588 clones were randomly selected and sequenced, resulting in 22 OTUs with the cut-off value of 97% nucleotide similarity as well (Table 2). The coverage (C) ranged from 56 to 83% in AOA and 73 to 93% in AOB (Figure 2). Rarefaction analysis indicated that AOA contained higher diversity than AOB (*P* < 0.01, *t*-test; Figure 2). For AOA, sample I704 had the highest diversity indices (H and 1/D) while the lowest indices occurred in sample I503; for AOB, the highest diversity indices occurred in sample I202 from the BOB and sample I501A contained the lowest one. Together with the richness estimator S_chao1_, higher diversity of AOA samples occurred in transections I4 and I5, while higher AOB diversity occurred in transections I1 and I2.

**Table 2:** **Diversity and predicted richness of archaeal and bacterial *amo*A sequences recovered from the EIO and SCS**.

**Sites**		**I506**	**I503**	**I501A**	**I412**	**I704**	**I207**	**I202**	**I105**	**I103**	**SCS**
AOA	No. of clone sequenced	43	59	88	78	60	44	88	50	50	90
	No. of OTUs	17	26	27	32	10	18	28	19	14	19
	Coverage (C%)	60.47	55.93	69.32	58.97	83.33	59.09	68.18	62.00	72.00	78.89
	Shannon–Weiner	2.28^**^	2.88^**^	2.34^**^	2.54^**^	1.26	2.37^**^	2.73^**^	2.34^**^	2.32^**^	1.87^*^
	1/D	6.74^*^	15.8^**^	5.15	5.15	2.18	7.65^*^	9.79^**^	6.92^*^	8.86^**^	3.15
	S_chao1_	35.33	60.00^**^	69.75^**^	87.2^**^	13.33	57.00^**^	43.00^*^	45.00^*^	16.00	23.67
AOB	No. of clone sequenced	78	79	20	94	90	55	44	41	42	45
	No. of OTUs	11	9	4	13	6	14	10	11	5	7
	Coverage (C%)	85.90	88.61	80.00	86.17	93.33	74.55	77.27	73.17	88.10	84.44
	Shannon–Weiner	1.87^**^	1.08	0.71	1.73^**^	0.85	2.21^**^	2.24^**^	2.04^**^	0.94	1.48^*^
	1/D	4.70^*^	1.88	1.56	3.26	1.73	7.00^**^	10.00^**^	7.07^**^	1.90	3.75
	S_chao1_	11.25^*^	12.00^*^	4.50	13.00^**^	6.50	15.20^**^	14.00^**^	21.00^**^	5.00	7.33

*Unique OTUs of the amoA sequences were determined using the DOTUR program. The coverage(C), Shannon–Weiner (H), Simpson (D) and S_chao1_ richness estimators were calculated based on the OTU data*.

Differences between data set of each index are determined by t-test, significance is represented by P-value.

* P < 0.05;

** *P < 0.01*.

### Phylogeny of archaeal and bacterial *amo*A genes

**Figure 3:**
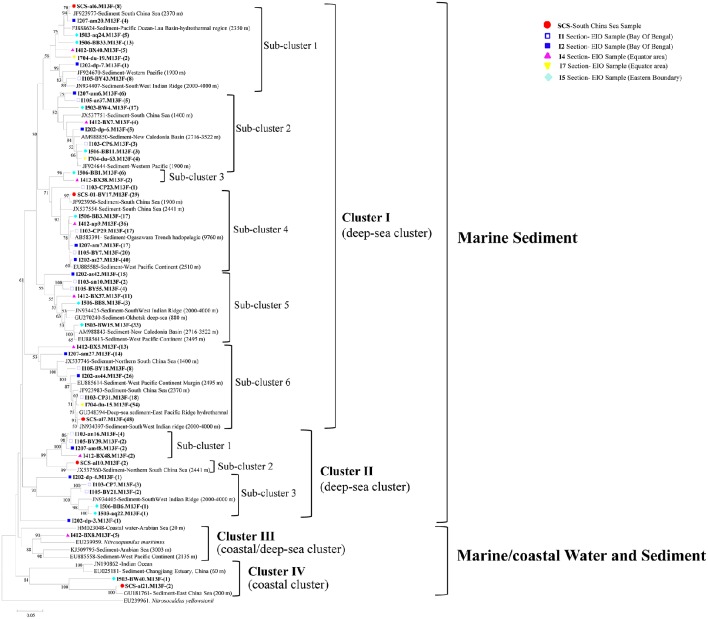
**Reconstructed phylogenetic tree with distance and neighbor-joining method of partial archaeal *amo*A sequences recovered from the sediments of the EIO and the SCS**. The tree branch distances represented the substitution rate and the scale bar represented the expected number of changes per homologous position. Bootstrap values >50% of 1,000 resamplings were shown near nodes. The archaeal *amo*A sequences obtained in this study were shown with colored labels along with the site, and clone numbers for each OTU were included in brackets.

The 53 representative archaeal *amo*A gene sequences shared 72.8–96.9% similarity among each other and 92–99% identities to the closely matched sequences from GenBank. The top hit sequences in GenBank were mainly retrieved from deep-sea environments, including sediments from the SCS (Cao et al., 2012), the West Pacific Margin and Continent (Luo et al., 2015), the Ogasawara Trench hadopelagic (Nunoura et al., 2013), the New Caledonia Basin deep subsea-floor (Roussel et al., 2009), hydrothermal vent at the Mid-Atlantic Ridge (Xu et al., 2014), the South West Indian ridge, and the East China Sea (Dang et al., 2010). Sequences obtained from deep-sea water column from the Mariana Trench (Nunoura et al., 2015), the Indian Ocean, coastal water, and sediment from the Arabian Sea were also included (Figure 3). No clones correlated with terrestrial or freshwater environment were detected, and most of the clones were originated from the marine sediment with a depth ranging from 1,400 to 9,760 m.

All of the archaeal *amo*A sequences formed two big groups: Marine sediment, and Marine/coastal water, and sediment. Within the two groups, four clusters with nine sub-clusters were identified in the phylogenetic tree (Figure 3). In Cluster I, *amo*A sequences were mainly associated with deep-sea sediments. Six sub-clusters were distinguished within cluster I, among which, three of them included clones from the EIO exclusively; while the rest of them contained clones from both the EIO and the SCS together (Figure 3). In Cluster II, three sub-clusters were defined. Sub-cluster 1 contained clones from the BOB (I103, I105, and I207) and the Equator (I412-B48); sub-cluster 3 contained clones from the BOB and the Eastern Boundary only. Two clones from the SCS (SCS-al10) were grouped with a sequence from the Northern SCS (JX537560) and formed sub-cluster 2. In Cluster III, only clones from the Equatorial area (I412-BX8) were presented. Within Cluster IV, two clones from the SCS (SCS-al21) and one clone from the Eastern Boundary (I503-BW40) were grouped together. One unique clone from site I202 (I202-dp-4) formed a single clade and represented a standalone clade from the BOB sediment. Among all the clusters, sub-cluster 4 and sub-cluster 6 within Cluster I contained most of the clones and accounted for 23 and 25% of the total sequences (Figure 3).

**Figure 4:**
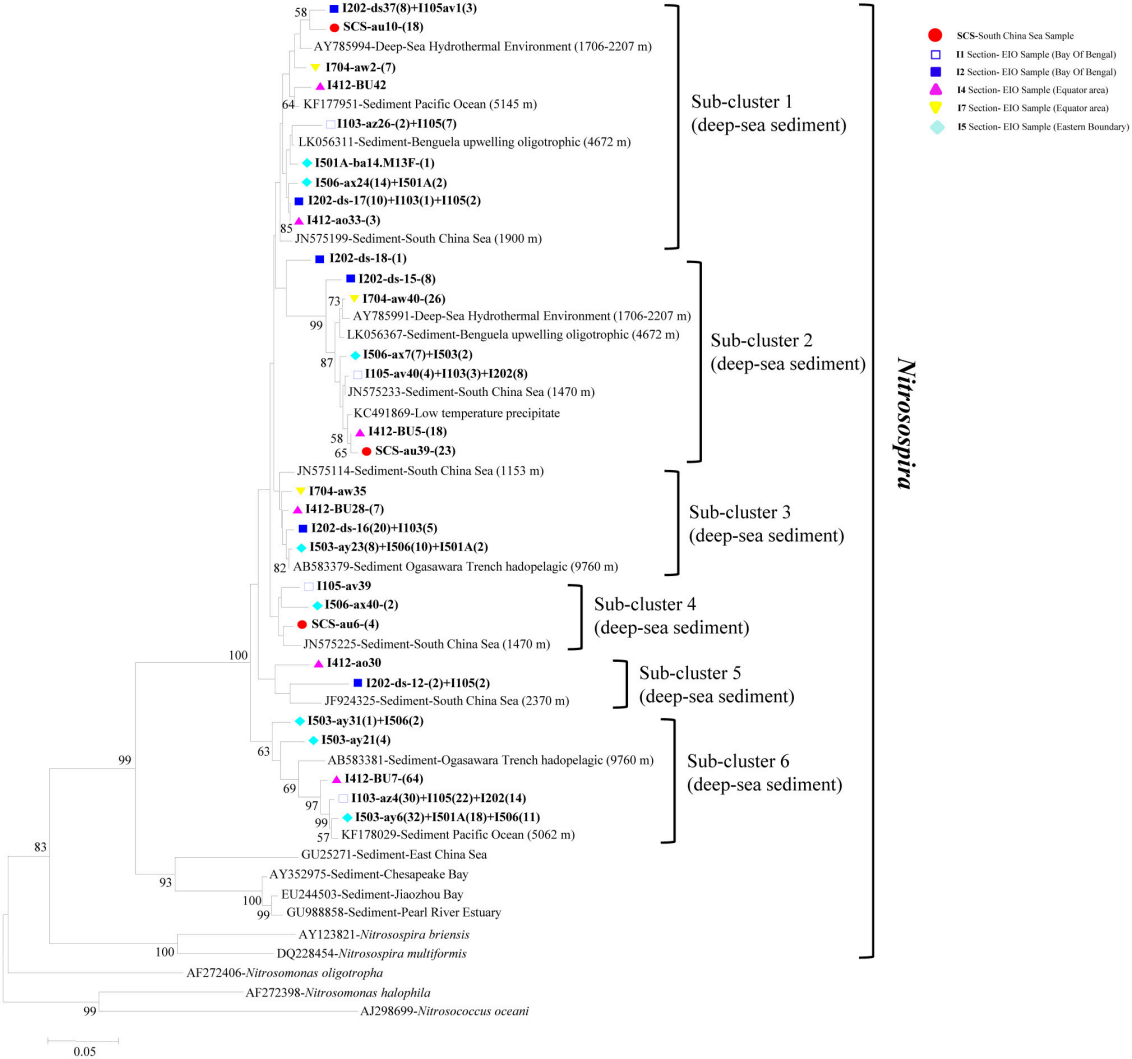
**Reconstructed phylogenetic tree with distance and neighbor-joining method of partial bacterial *amo*A sequences recovered from the sediments of the EIO and the SCS**. The tree branch distances represented the substitution rate and the scale bar represented the expected number of changes per homologous position. Bootstrap values >50% of 1,000 resamplings were shown near nodes. The bacterial *amo*A sequences obtained in this study were shown with colored labels along with the site, and clone numbers for each OTU were included in brackets.

In phylogenetic analysis of AOB, the 30 representative bacterial *amo*A sequences shared 87.3–95.9% similarities among each other and quite high identities to the closest match sequences from GenBank with a similarity of 97–99% (Figure 4). The top hit sequences blasted in GenBank were from deep-sea sediments, such as the Pacific Ocean deep-sea sediments (Luo et al., 2015), the SCS Sediments (Cao et al., 2012), hadopelagic sediments in the Ogasawara Trench (Nunoura et al., 2013), oligotrophic surface sediments of the Benguela upwelling system, low-temperature hydrothermal Fe-Si-rich precipitates, and deep-sea hydrothermal sediments. All bacterial *amo*A genes obtained in this study belonged to *beta*-proteobactetria (*Nitrosospira*); no clones were grouped with *Nitrosomonas* or *Nitrosococcus*. Within the big group of *Nitrosospira*, six sub-clusters were identified with their closely aligned sequences from GenBank. As shown in Figure 4, sub-cluster 1 and sub-cluster 2 were the two largest sub-clusters. Clones retrieved from this study were presented in all six sub-clusters with clone numbers in sub-clusters 1 and 2 higher than the other four sub-clusters. Clones in sub-cluster 3 were associated with sequences from the EIO mainly while sub-cluster 4 contained sequences from site I105, I506, and the SCS. Sub-cluster 5 and sub-cluster 6 contained sequences from transections I5, I4, I1, and I2, which were all located at the EIO.

### Archaeal and bacterial *amo*A gene abundance

**Figure 5:**
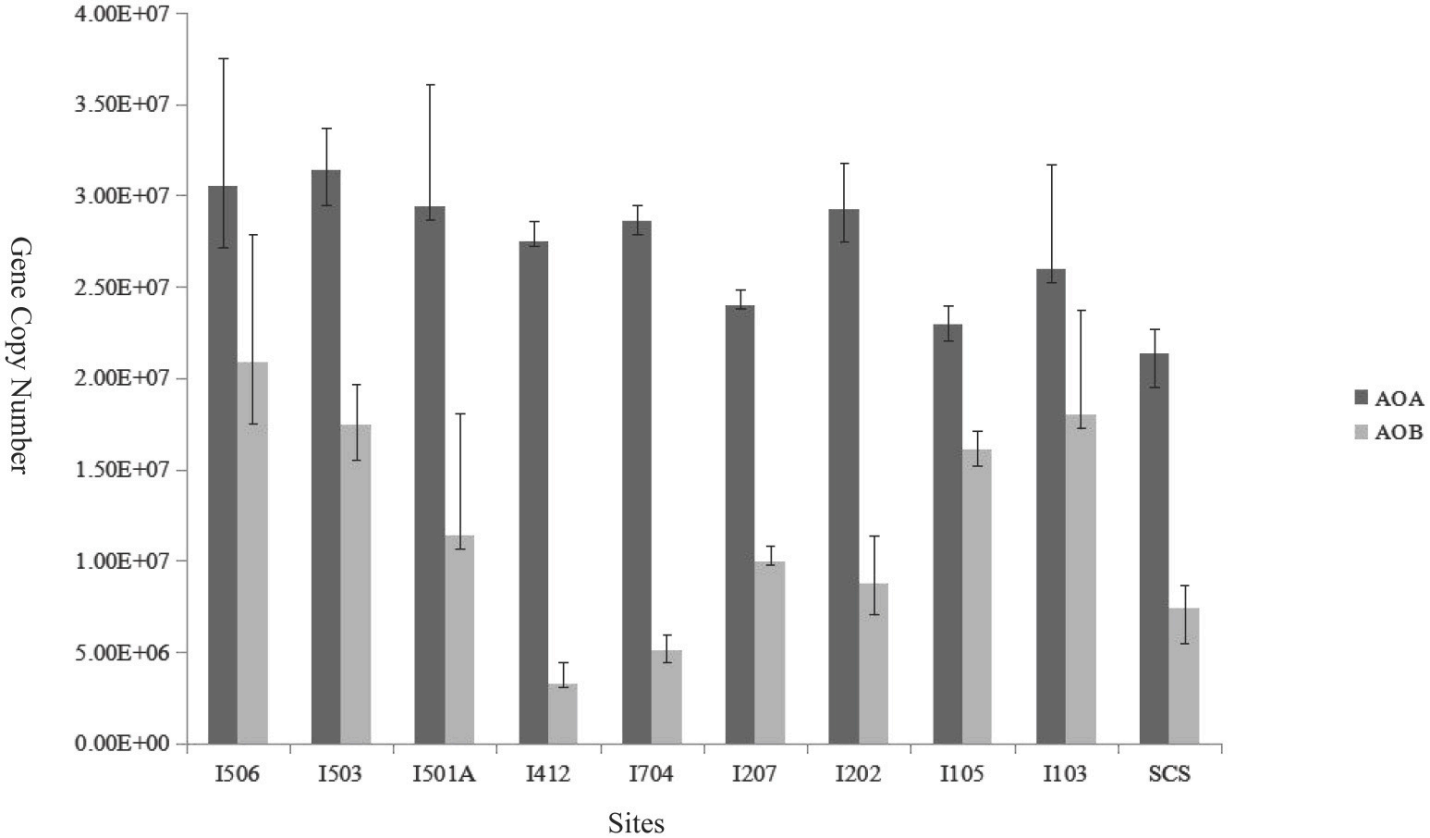
**Relative abundance (copies per gram of wet weight sediment) of archaeal and bacterial *amo*A genes in the deep-sea sediment of the EIO and the SCS**. *Error bars* represented the standard deviations of the independent triplicate qPCR reactions.

The copy numbers of AOA *amo*A genes ranged from 2.14 × 10^7^ to 3.14 × 10^7^ copies/g sediment (wet weight), while AOB *amo*A gene copy numbers ranged from 3.27 × 10^6^ to 2.08 × 10^7^ copies/g sediment (wet weight; Figure 5). Ratios of AOA/AOB *amo*A gene abundance ranged from 1.42 to 8.49. The gene copy numbers of AOA were significantly greater than that of AOB in every sample of the studied sites (*t*-test, *P* < 0.01). Among the 10 samples, AOB *amo*A gene abundance at site I412 was lower than all the other sites (*t*-test), and sites I103, I105, I503 and I506 were higher compared to the rest six samples. In general, AOB *amo*A genes shared bigger variability among the samples than AOA.

### Distribution patterns of *amo*A genes and potential environmental drivers

**Figure 6:**
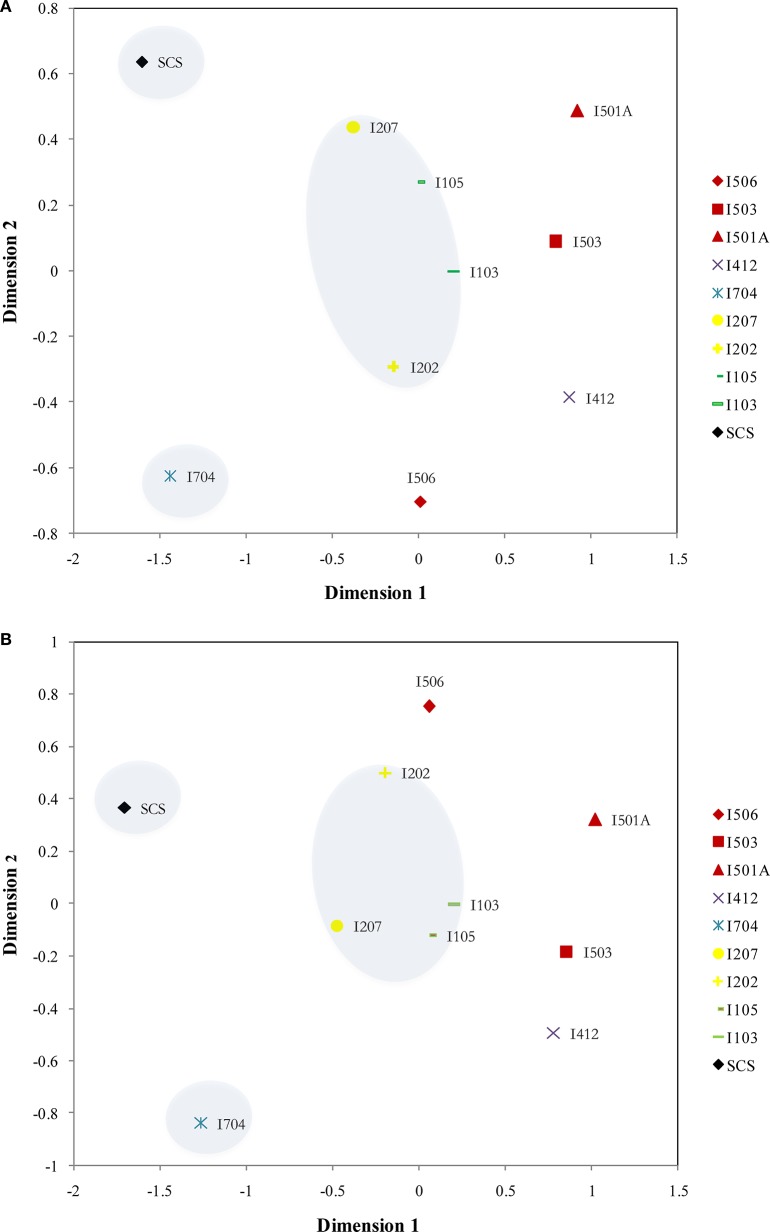
**NMDS ordinations of the archaeal *amo*A (A)** (Stress = 0.076) and bacterial *amo*A **(B)** (Stress = 0.084) assemblages from the sediments of the EIO and the SCS. Samples from the same transections were indicated by color.

Distribution patterns of AOA and AOB were similar based on the NMDS results: SCS and I704 were distinct from all the others, and samples from the BOB (I103, I105, I207, and I202) were grouped together (Figure 6). The samples from the Equator area (I4) and the Eastern Boundary (I5) showed similarity with the samples from the BOB, and also demonstrated the spatial differences along the transect (I5).

**Figure 7:**
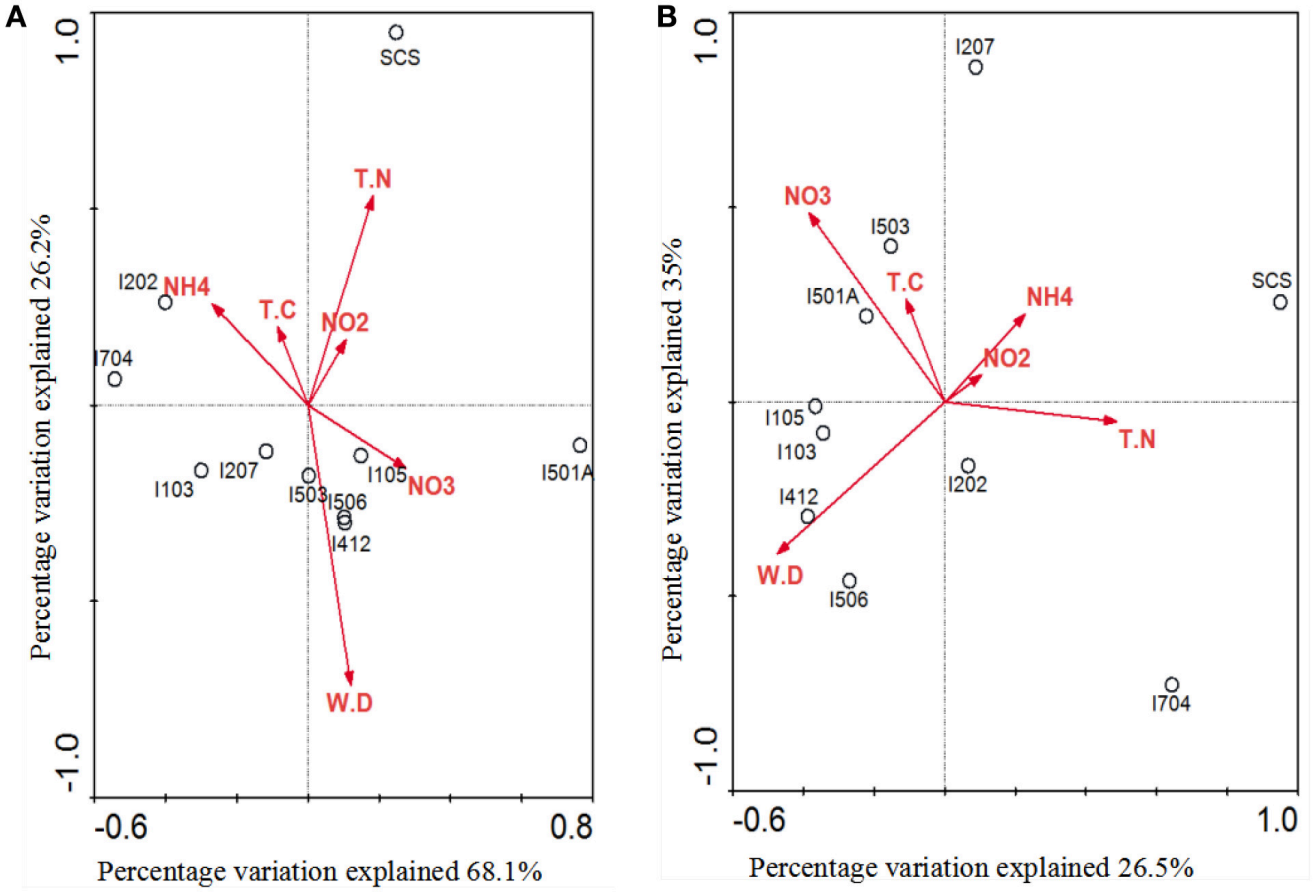
**RDA ordination plots for the relationship between the AOA (A)** and AOB **(B)** community distributions with the environmental parameters in the deep-sea sediments of the EIO and the SCS. Correlations between environmental variables and RDA axes were represented by the length and angle of arrows (environmental factors). TN, total nitrogen; TC, total carbon; WD, water depth.

Correlations of AOA/AOB community structures and the associated environmental variables were analyzed by multivariate analysis RDA (Figure 7). The environmental variables in the first two RDA dimensions explained 94.3% of the total variance in the archaeal *amo*A genotype composition and 61.5% of the total variance in the bacterial *amo*A genotype composition. Results indicated that AOA community structure in the sediments of EIO responded to water depth, ammonium and nitrate concentration, and these three factors provided 33.4, 22.3, and 19.7% of the total RDA explanatory power. Ammonium contributed significantly to archaeal *amo*A gene distributions (*P* = 0.047, 533 Monte Carlo permutations). However, the community structure from SCS was more strongly correlated with concentration of total nitrogen and nitrite (Figure 7A). For AOB, nitrate concentration and water depth contributed to the distributions of bacterial *amo*A genotype, which provided 25 and 14.8% of the total RDA explanatory power, respectively. The combination of other variables provided additionally 21.7% of the total RDA explanatory power (Figure 7B), but none of the parameters showed significant correlations.

## Discussion

Deep-sea floor sediment constitutes the largest land on earth. It is characterized by extreme conditions including high pressure, low temperature, lack of light etc., and it harbors taxonomically and metabolically diverse microorganisms (D'Hondt et al., 2004; Inagaki et al., 2006; Kallmeyer et al., 2012). Among those microbes, bacteria dominated over archaea in the oxic surficial sediments, and the dominant groups varied with environmental gradients (Orcutt et al., 2011). More recently, high throughput sequencing data showed similar observations in the largest oceanic oxygen minimum zone (OMZ)—the North Pacific Ocean (Beman et al., 2012b), Arctic deep-sea sediments (1,200–5,500 m water depth; Rapp et al., 2016), and the marginal region of the Indian Ocean (Wang et al., 2016). However, as Orcutt et al. (2011) pointed out, compared to other regions, our knowledge of deep ocean sediment is still scarce. Based on the authors' knowledge, detailed community composition and distribution patterns of nitrogen transforming microorganisms in the EIO have not been explored to date.

Our data showed a dominance of AOA over AOB within the ammonia-oxidizing community and the ratios of AOA/AOB *amo*A genes ranged from 1.42 to 8.49. This observation corroborated the previous results in most deep ocean sediments including the SCS, the Ogasawara Trench, the Northwest Pacific Ocean, and the Mariana Trench (Beman et al., 2012a; Dang et al., 2013; Nunoura et al., 2013; Luo et al., 2015). However, a few studies showed the opposite trend, where the dominance of AOB over AOA was found in the Mid-Atlantic Ridge of the South Atlantic Ocean sediments (Xu et al., 2014) and in surface sediments in the oligotrophic South Atlantic Gyre (Lagostina et al., 2015). The inconsistent results suggest that further investigations are required to explore the distribution and function of these ammonia oxidizers in deep ocean environments. We summarized and compared the diversity and abundance of archaeal and bacterial *amo*A genes in Table 3, including the results from this study and also other deep ocean environments. In general, OTU numbers of AOA were greater than AOB in the sediments from deep oceans such as the Pacific Ocean, the South Atlantic Ocean, the South China Sea, the Ogasawara Trench, and the EIO (Table 3). Among the eight studies, the abundance (gene copy number) of archaeal *amo*A genes was greater than bacterial *amo*A genes in five of them, with water depths ranging from 3,386 to 5,725 m. In contrast, the OTU numbers of AOB were higher than AOA in the shallower environment, like the Gulf of Mexico (Table 3). However, we are also aware that the number of sequences/reads (sequencing depth) varied in each study, which make the comparisons not straightforward. Another impacting factor is the potential PCR bias. Since identical primers were used to amplify the *amo*A genes among these studies, we assumed the PCR bias would be minimal and it is possible for us to make these direct comparisons.

**Table 3:** **Summary of the abundance and phylogenetic diversity of AOA and AOB in deep-sea environments**.

**Ocean environment**	**Resources**	**Depth (m)**	**Abundance**	**No. of OT Us (No. of clones sequenced)**		**Phylogenetic affinity (AOA branch)**	**Phylogenetic affinity (AOB branch)**	**References**
			**AOA vs. AOB**	**AOA**	**AOB**			
Ogasawara Trench	Sediment	9760	AOA > AOB	26 (117)	8 (133)	Related to marine water and sediments	Marine environment	Nunoura et al., 2013
Northwest Pacific Ocean, Mariana Trench	Sediment	5017–7068	AOA > AOB	48 (704)	14 (801)	Marine sediment	Deep sea environment	Luo et al., 2015
Central Pacific Ocean	Sediment	5062–5145	AOA < AOB					
South Atlantic Gyre (SAG)	Sediment	1944–4672	AOA < AOB	12 (258)	6 (264)	Marine environment	Marine environment	Lagostina et al., 2015
Mid-Atlantic Ridge (MAR) of the South Atlantic Ocean	Sediment	2721–2807	AOA < AOB	14 (472)	2 (457)	Water column/sediments	Deep sea environment	Xu et al., 2014
West Pacific Continental Margin	Sediment	1390–3520	–	83 (735)	–	Marine, terrestrial or estuarine environments	–	Dang et al., 2009
Northeastern Japan Sea	Sediment	2000–2956	AOA > AOB	16 (40)	9 (14)	Deep marine and sediment	Deep-ocean	Nakagawa et al., 2007
Northern South China Sea	Sediment	1050–2456	AOA > AOB	131 (1457)	–	Mainly marine environment	–	Dang et al., 2013
Pearl River Estuary to the South China Sea	Sediment	2370	AOA > AOB	19 (60)	11 (39)	Marine environment	Marine and estuarine environments	Cao et al., 2011b
Hydrothermal vents of the Pacific Ocean	Sediment	2192–2267	–	33 (93)	–	Water column/sediments, soils	–	Wang et al., 2009
Hydrothermal vents of the Pacific Ocean	Sediment	1370–1385	–	13 (120)	–	Seawater and ocean sediment	–	Nunoura et al., 2010
Gulf of Mexico	Sediment	1300	–	9 (45)	12 (46)	*Nitrosopumilus maritimus*	*Nitrosospira/Nitrosomonas*	Flood et al., 2015
The Eastern Indian Ocean	Sediment	3386–5725	AOA > AOB	87 (650)	30 (588)	Deep sea sediment	Deep sea sediment	This study

Dominance of AOA in the deep EIO sediment indicated that Archaea may adapt to low oxygen and low nutrient environments. Concentrations of [${\text{NO}}_{2}^{-}$ + ${\text{NO}}_{3}^{-}$] and ${\text{NH}}_{4}^{+}$ in our samples ranged from 0.002 to 0.0045 mg/L and 0.0019 to 0.0045 mg/L, respectively, which were lower than that in the Pacific Ocean and the shallow SCS (Cao et al., 2011a; Dang et al., 2013). Although AOB seems to have at least 10-fold higher cell activity than those of AOA, AOA appeared to be more sensitive to ammonia than AOB (Prosser and Nicol, 2012). Simulations based on experimental data from cultured AOA and AOB strains suggested that AOA grow faster than AOB at lower ammonia concentrations (Prosser and Nicol, 2012). Indeed, AOA have kinetic advantages over AOB under low substrate concentrations due to their up to 200-fold higher affinity for ammonium (Martens-Habbena et al., 2009, 2015). Furthermore, Archaea have developed low-permeability membranes in order to facilitate catabolic pathways, by which they are able to thrive under energy stress (Valentine, 2007). Moreover, deep-sea Archaea even might recycle membrane lipids between growing cells and the surrounding sediment in order to save energy (Takano et al., 2010). All the above evidences indicate the fact that Archaea could outcompete Bacteria or even phytoplankton in oxidizing ammonia under nutrient-limited conditions, such as the oligotrophic deep-sea sediments of the EIO.

All the archaeal *amo*A gene sequences from this study were clustered within the group of marine sediment, consisting of previously reported AOA sequences from the West Pacific Continental Margin, the SCS, and others (Figures 3, 4). No AOA clones were grouped into the clusters affiliated with estuarine or terrestrial environments. Meanwhile, the AOB sequences identified from the current study were all related with *Nitrosospira*-like species, which were commonly found in high salinity environments (see Section Discussion below). All these indicated the pure marine origin of the AOA and AOB clones revealed from this study. What's more, we did not observe distinct separation of the SCS sample from the EIO samples by forming unique clusters containing clones from the SCS only, with the exception of one small subcluster (Cluster II, Sub-cluster 2) containing two SCS clones in the AOA phylogenetic tree (Figure 3). We did observe distinct EIO clusters in both AOA and AOB phylogeny (Figures 3, 4). The Indian Ocean seemed to harbor large amount of local species of AOA and AOB in the sediments. In addition, due to the location specialization, novel or unique microorganisms were also expected to be present in deep EIO sediments. In our study, defined with 97% similarity, 5.8% of the archaeal and 3.6% of the bacterial *amo*A clones were previously un-revealed environmental sequences, suggesting the possible existence of unique ammonia-oxidizing microbes in the EIO sediments.

As expected, our data also indicated that the archaeal *amo*A genes contained higher diversity than bacterial *amo*A genes (Table 2). More variations as well as greater divergences of AOA genotypes than AOB sequences were observed from the constructed phylogeny (Figures 3, 4). High diversity of AOA has been observed from deep-sea sediments in the Pacific Ocean (5,017–7,068 m), the SCS (up to 2,370 m) and the northeastern Japan Sea (2,000–3,000 m; Nakagawa et al., 2007; Cao et al., 2011b; Luo et al., 2015; Table 3). However, in sediments from the Coastal North Sea, the SCS and the Mariana Trench, AOA was more abundant than AOB but gene variation of AOA was less significant than AOB (Wuchter et al., 2006; Cao et al., 2011b; Luo et al., 2015). Some previous studies also demonstrated that AOA and AOB were quite stable in terms of *amo*A gene copy numbers and both showed equal levels of variation (Mosier and Francis, 2008; Park et al., 2008; Jin et al., 2011; Zheng et al., 2013; Lagostina et al., 2015). Nevertheless, the disagreement of abundance and genetic variations between AOA and AOB distributions strongly suggested these two types of microbes are highly adapted to local environments and therefore their population dynamics are likely driven by ambient environmental gradients.

Our RDA analyses did not show significant correlations between total carbon and AOA/AOB distributions, but ammonia, nitrate and total nitrogen influenced the distribution of nitrogen transforming microbes (Figure 7). Distribution patterns inferred by NMDS showed a good separation of communities from the BOB and those close to the Equator; and the SCS was also separated from sites in the EIO (Figure 6). The distribution patterns revealed in this study were in certain accordance with the observations that presumably the environmental gradients including organic matter, nutrients and salinity impact the distribution and abundance of ammonia oxidizers (Urakawa et al., 2006; Dang et al., 2009; Bernhard and Bollmann, 2010; Wang et al., 2014; Xie et al., 2014). At the same time, AOA or AOB also responded differently to the environments. For example, Archaea tended to be less sensitive to the changes of nutrient availability (Leininger et al., 2006; Zheng et al., 2013), while the abundance of Bacteria responded to total carbon or ammonium concentrations (Zheng et al., 2013; Luo et al., 2015; Lagostina et al., 2015). Taken the above two points into consideration, likely the nutrient gradients (e.g., nitrate and ammonium in this study) between the BOB and the Equatorial area influenced the distribution patterns of nitrogen transforming microorganisms in the EIO sediments. Moreover, the total carbon (TC) ranged from 5.56% in the Equator (average of sites I412, I704, and I501A) to 10.87% in the BOB (average of sties I103, I105, 1202, and I207), which might also have contributed to the separation of these two groups of samples.

A close correlation of archaeal and bacterial *amo*A community distribution with water depth was also observed in our study (Figure 7). As reported, geographic location, light, oxygen concentration, and water depth were believed to influence vertical structure and richness of marine microbes in general (Walsh et al., 2014). Based on the nomenclature of AOB, all of our clones fell into deep-sea marine sediments cluster (Avrahami and Conrad, 2003), and were associated with high salinity and low ammonium environments (Dang et al., 2010). Ammonia oxidizers have been found to be associated with salinity gradients (Bernhard et al., 2005, 2007): *Nitrosospira* was more abundant in high-salinity environment, while *Nitrosomonas* preferred low-salinity environments (Dang et al., 2010; Jin et al., 2011). In most cases *Nitrosospira*-like species were dominant in marine environments (Bernhard and Bollmann, 2010), such as in deep-sea environment of Japan Sea, Atlantic Ocean, and Pacific Ocean (Nakagawa et al., 2007; Xu et al., 2014). Results from this study further strengthen this view by showing a preliminary dominance of *Nitrosospira* over *Nitrosomonas* in the deep-sea sediment of EIO (Figure 4).

Results from this study provided insights of diversity and distributions of AOA and AOB in the oligotrophic EIO sediments. Diversity and abundance of AOA outnumbered AOB in each sample, and water depth and nutrient gradients (e.g., [NO_2^−^_ + NO_3^−^_], NH_4^+^_ concentration) were the main driving factors in shaping the distribution patterns from the Equator to the BOB. However, in natural settings, it is difficult to identify single environmental factors that explain the relative abundance of AOA and AOB. As Prosser and Nicol (2012) pointed out, further investigations on niche specialization and differentiation of AOA and AOB are needed, including in the BOB where the oxygen concentration is lower than other areas of the EIO. Future studies of these aspects will shed light on links between microbial distribution and nitrogen cycling in global oceans.

## Author contributions

This work was designed by JS, JW, and JK. Samples were collected by JW. Experiments were carried by JW, XDZ, XCZ, ZX, and XL. Data were analyzed by JW, YM, and GQ. The manuscript was drafted by JW and revised by JK.

## Funding

This work was supported by the Natural Science Foundation of China under contract Nos. 41276124 and 41506182, and Endowment from Stroud Water Research Center; Science Fund for University Creative Research Groups in Tianjin under contract No. TD12-5003; the Program for Changjiang Scholars to JS; the Science Fund from the Tianjin University of Science & Technology to JW under No. 0001001.

### Conflict of interest statement

The authors declare that the research was conducted in the absence of any commercial or financial relationships that could be construed as a potential conflict of interest.
